# Effectiveness of an Ecological Model-Based Active Transport Education Program on Physical and Mental Health in High School Students (MOV-ES Project): Study Protocol for a Randomized Controlled Trial

**DOI:** 10.3390/healthcare12131259

**Published:** 2024-06-25

**Authors:** Eugenio Merellano-Navarro, Andrés Godoy-Cumillaf, Daniel Collado-Mateo, Mirko Aguilar-Valdés, Jorge Torres-Mejías, Alejandro Almonacid-Fierro, Pablo Valdés-Badilla, Frano Giakoni-Ramírez, José Bruneau-Chávez, Pedro R. Olivares

**Affiliations:** 1 Department of Physical Activity Sciences, Faculty of Education Sciences, Universidad Católica del Maule, Talca 3530000, Chile; aalmonacid@ucm.cl (A.A.-F.); valdesbadilla@gmail.com (P.V.-B.); 2 Grupo de Investigación en Educación Física, Salud y Calidad de Vida (EFISAL), Facultad de Educación, Universidad Autónoma de Chile, Temuco 4780000, Chile; andres.godoy@uautonoma.cl; 3 Sport Sciences Research Center, Rey Juan Carlos University, Fuenlabrada, 28943 Madrid, Spain; danicolladom@gmail.com; 4 Faculty of Education Sciences, Universidad Católica del Maule, Talca 3530000, Chile; mirko.aguilar@alu.ucm.cl; 5 Doctoral Program in Physical Activity Sciences, Faculty of Education Sciences, Universidad Católica del Maule, Talca 3530000, Chile; jorge.torres.02@alumnos.ucm.cl; 6 Sports Coach Career, School of Education, Universidad Viña del Mar, Viña del Mar 2520000, Chile; 7 Faculty of Education and Social Sciences, Universidad Andres Bello, Las Condes, Santiago 7550000, Chile; frano.giakoni@unab.cl; 8 Departamento de Educación Física, Deportes y Recreación, Universidad de la Frontera, Temuco 4811230, Chile; jose.bruneau@ufrontera.cl; 9 Faculty of Education, Psychology and Sport Sciences, University of Huelva, 21007 Huelva, Spain; pedro.olivares@ddi.uhu.es

**Keywords:** active transport, adolescents, physical activity, school intervention, youth

## Abstract

The United Nations, through its 2030 Agenda and Sustainable Development Goals, advocates for the establishment of conducive environments for physical activity, following the ecological model. In line with this initiative, active transportation emerges as an accessible, cost-effective, and sustainable approach to augmenting daily physical activity levels. This study protocol endeavors to assess the impact of an active transportation education program rooted in the ecological model on the physical and mental well-being of high school students. Drawing upon scientific insights, we hypothesize that a 16-week active transportation intervention will lead to a 3% reduction in average body fat percentage and a noteworthy enhancement in executive function (including inhibition, cognitive flexibility, and working memory), physical fitness (comprising cardiorespiratory fitness and muscle strength), and mental health (encompassing mood disorders and cognitive functioning). If this intervention proves effective, it could offer a viable solution for the school community, especially in reducing congestion within the school environment. The study protocol aims to evaluate the impact of an active transportation educational program based on the ecological model on the physical and mental well-being of high school students. Three high schools located in the urban area of Talca, Chile, will be randomly selected (one public, one privately subsidized, and one private non-subsidized). Each high school will be randomly assigned an experimental group (n = 30) and a control group (n = 30; without intervention). The experimental groups will receive an active transportation educational intervention during their physical education classes for four months (60 to 90 min sessions, once a week), while the control group will receive no intervention. The primary outcome will provide information on body composition and executive function. Secondary outcomes will include objective physical activity level, physical fitness, mental well-being, academic achievement, health-related quality of life, perception of environmental urban features, physical activity barriers, and adherence to active transportation. It is expected that the results of the MOV-ES Project will transcend the physical health of schoolchildren and will have an impact on the school community, especially by decongesting the school environment.

## 1. Introduction

Physical inactivity is the fourth most important risk factor for mortality worldwide, and it is responsible for the deaths of five million people per year, making it a global public health priority [1,2]. Approximately 80% of adolescents fail to meet international physical activity recommendations, which state that ≥60 min of moderate to vigorous physical activity should be performed every day [3]. Given this problem, the World Health Organization (WHO), through the “Global Action Plan on Physical Activity 2018–2030”, proposes to reduce physical inactivity worldwide by 10% by 2025 and 15% by 2030 through initiatives to promote physical activity that involve a systemic view of the problem and that consider the school system as a relevant actor [4].

The importance of promoting physical activity at school age lies in the fact that it is in childhood and adolescence where the learning of healthy lifestyles, habits, and good living should be learned and strengthened [5,6,7], especially in adolescence (high school), as studies show that physical activity decreases during this phase [8,9]. Sustained physical inactivity behaviors lead to increased overweight/obesity in the school population [10,11], and mental health problems [12]. It is estimated that the cost associated with physical inactivity and obesity in Chile amounts to USD 103 million per year, with a direct cost of USD 69.2 million, of which 31.7% is borne by the family [13].

The WHO points out that one of the reasons for the low physical activity level at school age is the school itself, due to the long days of low energy expenditure within the school [14]. There is evidence that the times when there is most physical activity in school are before school (transportation), during recess, lunch, and after school [15]. In this overall scenario of physical inactivity, active transportation (walking or cycling to and from school) represents an opportunity for children to engage in regular physical activity [16], becoming an economical and sustainable option that impacts the daily living of schoolchildren [17,18,19,20,21]. A meta-analysis indicates that active transportation could contribute between 23% and 36% of daily physical activity in children and adolescents [22]. Other studies show that schoolchildren who walk or cycle to school achieve higher levels of physical activity [23], lower adiposity [20,24], better cardiorespiratory fitness [25], a higher level of happiness and well-being [26], and improved academic achievement [27] compared to schoolchildren who do not use it.

The rate of active transport use among schoolchildren varies from country to country [9], but in general, it is low and is steadily declining in more economically developed countries [28,29,30,31]. In Chile, there is a lack of studies to establish an objective rate of Chilean schoolchildren who use active transportation to school [32], which is evidenced in the latest Notebook (Global Matrix Chile) [11]. The incipient evidence indicates that only 8.9% of schoolchildren engage in active transportation [32], which is very different from the data presented by a Chilean government agency, where 18.1% of adolescents report daily active transportation [33]. However, both data are lower than those reported in cities such as Ottawa (35.5%), Bogotá (72.14%), Helsinki (81.1%), Cape Town (51.3%), and Baton Rouge (10.8%) [34]. These differences in percentages should be understood based on the country’s characteristics, which should be equivalent to the level of economic development and the need versus choice that people have to opt for active or motorized transport [35]. Some systematic reviews identify predictors of transportation in children, highlighting demographic characteristics, individual and family, school, and physical and social environmental factors [36,37]. Other studies have focused on parental barriers to active transportation, establishing a negative and significant association between traffic and personal safety within the neighborhood adjacent to the school [38]. A recent study affirms that parents’ perceptions of active transportation differ according to the distance of the home from the school [39]. In this context, the greater the distance, the lower the levels of social support, the greater the environmental barriers, and the greater the safety concerns regarding physical activity, walking to school, and, less consistently, bicycling to school [39].

Recent studies in the Chilean school population have analyzed the impact of various physical activity interventions [40,41,42]. However, active transportation interventions in the Chilean school population have not been explored. The evidence indicates that interventions that involve a socio-ecological view of the problem, including intrapersonal factors (biological and motivational), interpersonal (social) and community networks, and socioeconomic, cultural, and environmental conditions, benefit the student and their environment [43,44,45]. In this sense, the ecological model proposed by Sallis et al. indicates that active living behaviors are determined by the influence of intrapersonal, interpersonal, and physical environment factors, which specifically influence four domains of active living: occupation, transportation, domestic activities, and recreation [46]. By intervening in each of these, community physical activity levels can be increased. However, studies that establish objective data on physical activity and active transportation in Chile are lacking.

The effectiveness of active transportation interventions in the school setting has been widely studied [20,21,24,34]. The benefits of this type of intervention do not only include increased physical activity in schoolchildren but also increased positive emotions during the school trip [47], increased safety in the city [48], and improved academic achievement [49]. There is also evidence that promoting this type of transportation has an impact on the community [50,51], especially in the reduction in traffic congestion, exhaust, and greenhouse gas emissions [52,53,54]. Furthermore, although there are studies on active transportation in the Chilean population [32,33,55,56,57,58,59], there is a need for further studies focused on the school population.

At an international level, the work carried out at the University of Otago (New Zealand), through its BEATS Research program, stands out [50,60,61]. It is an interdisciplinary and multidisciplinary study based on the relationship between parents, academia, community, and government. Its purpose is to analyze the individual, social, environmental, and political influences on adolescents’ active transportation (walking or cycling) to school [60]. Within the project, some sub-studies yield cross-sectional [60] and longitudinal [50] data that support the proposal of the present study. In New York City, the Safe Routes to School program was implemented, increasing the rate of active transportation in the city and even reducing congestion and pollution [62]. In Spain, the University of Granada developed the “Pedalea y Anda al Cole (PACO)” project, which collected cross-sectional data on active transportation in different Spanish cities [63] and analyzed the effect of an educational intervention on active transportation to school by bicycle [64]. The PACO and PACA projects are currently being implemented; like the PACO Project, the intervention is educational and focuses on the use of bicycles [65].

Regarding the intervention duration, there is no consensus on how many weeks or months are best to increase physical activity through active transportation [64]. There are programs that intervene for one or two years [66,67], four months [68], or just a few weeks [69]. Most of the evidence that monitors and intervenes in the active transportation variable belongs to works from the United States of America [66,69,70], Spain [63,64], Denmark [71], the United Kingdom [72], Canada [67], Australia [73], and New Zealand [19]. However, there are no longitudinal studies in Latin America, which is relevant considering the differences with developed countries in terms of the school entry system, especially in Chile, where the variable “proximity to the home” may not be a determinant in the choice of the educational establishment [74].

From the call made by experts to design strategies contextualized to the local reality of active transportation to school [75,76] to increase physical activity levels and improve the physical and mental health of children, the project “Moving to School” (MOV-ES) was born. This project will be carried out during the period 2024–2026 in a city in Southern Chile that has urban characteristics below the national average in terms of population density (949.8 inhabitants/km^2^), sports facilities (0.81 m^2^/inhabitant), green areas (6.40 m^2^/inhabitant), and pollution problems above the national average [77]. Therefore, the aim of this study protocol for the MOV-ES project is to determine the effect of an active transport educational program based on the ecological model on the physical and mental health of high school students. Given the evidence from previous studies focused on reducing physical inactivity, it is hypothesized that (i) the MOV-ES intervention will result in a 3% decrease in mean percent body fat in the experimental group compared to the control group, and (ii) the MOV-ES intervention will result in a significant increase in executive function (inhibition, cognitive flexibility, and working memory), physical fitness (aerobic capacity and muscle strength), and mental health (mood disorders, cognitive functioning) in high school students.

## 2. Material and Methods

### 2.1. Study Design

**Figure 1:**
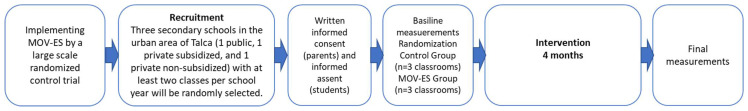
Flow diagram of the MOV-ES project.

This study includes an experimental design (randomized controlled trial), double blind, repeated measures, two parallel groups (one intervention and one control), and a quantitative approach. The methodology followed will be the Consolidated Standards of Reporting Trials Statement (CONSORT) methodology [78]. Figure 1 summarizes the study’s design.

### 2.2. Ethical Approval

The protocol of the current study adheres to the principles outlined in the updated Declaration of Helsinki and has received approval from the Human Ethics Committee of the Universidad Católica del Maule, Chile (approval number: N°27/2024). Prior to participation, all participants will provide written informed assent, and their parents will provide consent. Furthermore, the study has been registered in the Clinical Trial Protocol Registry and Results System (ClinicalTrials.gov) of the United States of America (code: NCT06357065; link: https://classic.clinicaltrials.gov/ct2/show/NCT06357065?cond=active+transport&draw=2&rank=1, accessed on 26 April 2024).

### 2.3. Sample Size Calculation

While feasibility trial results typically inform sample size calculations for scalability cluster randomized trials, an approximate sample size estimate is required for budgeting this large-scale trial. The sample size was determined to be 3 clusters (classrooms) per condition (n = 6 clusters) using the GRT sample size calculator from the NIH (researchmethodsresources.nih.gov). This calculation considered an absolute difference of 3% (alpha error of 0.05 and statistical power of 0.80) between the experimental group (EG) and control group (CG) in mean body fat percentage (with a standard deviation of 5), an average of 30 participants in each cluster, and a variance inflation factor of 1.05 based on prior research findings. Following the feasibility study and informed by qualitative research insights, adjustments will be made to refine the MOV-ES intervention.

### 2.4. Randomization and Blinding

All schools in the city of Talca, Chile, are electronically randomized to select three high schools located in the urban area of Talca (1 public, 1 private subsidized, and 1 private unsubsidized). Each high school will have an EG (n = 30) and a CG (n = 30; no intervention). This selection approach is in line with the Chilean educational system, which associates school administration with the socioeconomic level of families.

### 2.5. Participants

All first-year high school students (approximately aged 15 to 16 years old) from the chosen schools will be invited to participate in this study. Inclusion criteria for the students entail: (i) enrollment in the designated school; (ii) parental or legal guardian authorization (consent); (iii) do not have any motor problems to perform autonomous transport; and (iv) voluntary agreement from the students to take part in the study (assent).

### 2.6. Intervention

The CG will participate in the initial and final measurements but will not receive any intervention, attending their traditional physical education classes. On the other hand, the EG (MOV-ES) will receive an educational intervention within their physical education class. The contents of this intervention involve intrapersonal (benefits of active transportation and safety on the way to school), interpersonal (family safety), organizational, community, physical-environmental, and political (safety, infrastructure, and quality) factors [79]. The program will comprise a weekly session lasting 60 to 90 min each, spanning a total of 16 weeks. The entire intervention program was conducted during school hours within the framework of physical education classes and hosted on the premises of each respective institution. The curriculum will be structured into didactic units, which are divided into 16 subtopics that will be addressed in each of the sessions. Each session will have the following structure: (i) audiovisual presentation; (ii) application of content; and (iii) practical work. The contents will be complemented by the presentation of graphic and audiovisual materials together with practical components in the school space.The contents will follow the recommendations for active transportation interventions in schoolchildren [75] and the material available from the PACO Project (http://profith.ugr.es/pages/investigacion/recursos/manualbici, accessed on 20 June 2024). A summary of the progression and unit structure of the MOV-ES interventions is presented in Table 1.

The sessions of the last month of intervention will include practical activities in bicycle mechanics and walking in the school environment (outside the school). Depending on the possibilities of each school, cycling sessions will be included. At the end of the intervention, and to motivate students to engage in active transportation, both EG and CG participants will receive educational infographics.

### 2.7. Outcomes and Procedures

The research team has established partnerships with municipal corporations as well as public and private institutions through collaborative agreements. The project’s work plan will be consistently upheld throughout each year of its execution. At the beginning of each year, the randomization of establishments will take place. The research team will establish communication with the educational institutions, where they will introduce the study’s aims, duration, and key aspects of the data collection process to teachers and school administrators. Measurement days will be coordinated during this interaction. To inform and obtain consent from parents, the research team will employ various communication strategies: (i) request the school management unit to disseminate information about the study through available channels (e.g., emails, websites, notice boards); and (ii) arrange either virtual or in-person meetings with parents of each class (parent-teacher meetings), during which researchers will address inquiries about the research process and invite parents to complete the parental questionnaire. Informational materials about the study will be provided to the school for parents who do not have email access or could not attend the informational session. Parents will have the option to provide consent either in writing or electronically (recorded). Upon obtaining parental consent, student measurements will be scheduled. A battery of questionnaires and physical tests, based on the ecological model of physical activity and active transportation, will be administered, covering intrapersonal, interpersonal, organizational, community, physical-environmental, and political dimensions (pre- and post-intervention).

All the assessments will be carried out during the physical education class time, either in the classroom or at the designated location for each class. The research team will inform students about the project’s aims using informational materials; upon obtaining their assent, students will receive a kit containing an informational document about the study, the battery of school questionnaires, and the parental informed consent form. For this purpose, the research team will bring all the available material. It is estimated that the application of the questionnaire and the anthropometric measurements will last 3 h for each class measured (2 physical education class sessions).

#### 2.7.1. Primary Outcomes

The primary variables and the selected instruments for their measurement are summarized in Table 2. In addition to sociodemographic variables (such as birth date and gender), the following information will be collected from each participant at baseline:

Body composition: The percentage of fat mass and fat-free mass will be obtained using eight electrodes of tetrapolar bioimpedance (InBody 570^®^, Body Composition Analyzers, Seoul, Republic of Korea). Prior to the assessments, students will be asked to come to the measurement in light clothing, without metal-containing garments, and with the possibility of removing their footwear. This whole process will be carried out under the supervision of a school teacher.

Executive function: The NIH Toolbox for Assessment of Neurological and Behavioral Function^®^ (NIH Toolbox^®^ in Spanish, v. 1.8) was created as a comprehensive set of neurobehavioral assessments intended to rapidly evaluate sensory, motor, emotional, and cognitive abilities throughout the lifespan, spanning ages 3 to 85 years, available in both English and Spanish [80]. Inhibitory control and attention will be measured using the Flaker Test of Attention and Inhibitory Control. Cognitive flexibility and attention will be measured using the Dimensional Change Card Sorting Test, and working memory will be measured using the List Sorting Working Memory Test. For each test, the evaluator will read the specific instructions of the test to be applied, making sure, by verbal confirmation, that the subject understands the procedure. The results of the tests are extracted by means of scores.

#### 2.7.2. Secondary Outcomes

Anthropometric measurement: the following measurements are included: (i) body mass in kilograms using a digital scale (Seca 769, Hamburg, Germany; Accuracy of 0.1 kg); (ii) standing height in centimeters measured with a stadiometer (Seca 220, Hamburg, Germany; Accuracy of 0.1 cm); (iii) six diameters (biacromial, transverse thorax, anteroposterior thorax, bi-iliocrestid, humeral, femoral) in centimeters using an anthropometer (Rosscraft Campbell 20, Richmond, BC, Canada; Accuracy of 0.1 mm); (iv) ten circumferences (head, relaxed arm, arm under tension, maximum forearm, mesosternal thorax, minimum waist, maximum hip, maximum thigh, medial thigh, maximum calf) in centimeters measured with an inextensible measuring tape (Seca 201, Hamburg, Germany; Accuracy of 0.1 cm); and (v) six skin-fold thicknesses (triceps, subscapular, supraspinal, abdominal, medial thigh, calf) in millimeters assessed using a caliper (Harpenden, Hitchin, UK; Accuracy of 0.2 mm). According to the International Society for the Advancement of Kinanthropometry (ISAK) [81], all assessments will be performed by an ISAK level II anthropometrist.

Physical activity level (objective): Measurement of objective physical activity level will be by Actigraph Accelerometer—Model wGT3X-BT, Pensacola, FL, USA. They include a microelectromechanical system (MEMS)-based accelerometer, ambient light, and a touch sensor as data collection endpoints. The study subjects will wear the device for 6 full days [82]. At the end of the measurement period, the accelerometer will be removed by the technical staff of the research team for analysis. Sedentary time will be set at <1100 count/minute, light intensity between 1100 and 3200 count/minute, and moderate-vigorous intensity at >3200 count/minute. Accelerometry data will be valid if they present ≥3 days of measurement with a minimum of 9 h of use per day. The data obtained through the accelerometers will be further processed by the Actilife 6.13.4 software and the GGIR statistical package [83].

Physical fitness: It will be assessed with the application of different tests validated in the school population, with high reliability and ease of application. The order of application of the tests contemplated is: (i) A 20 m shuttle run test will be used to measure the maximal oxygen consumption (VO_2_ max). This test allows a very reliable and easy-to-use evaluation. This is a maximal test involving a continuous run between two lines separated by 20 m at the same time as the beeps are recorded [84]. The athlete’s score is the level and number of laps (20 m) achieved before he/she could not keep up with the recording. (ii) A long jump test with feet together will be used to measure lower body muscle strength [85]. This test allows for or assesses the explosive force (power) of the extensor muscles of the legs; the number of centimeters advanced, from the jump line to the edge closest to the jump line, of the foot that was farthest behind after the fall is recorded. (iii) The upper body muscle strength will be assessed with the hydraulic dynamometer (Camry, model EH101, Zhongshan, China), which allows evaluation of great reliability and easy application [86].

Mental well-being: This will be measured with the Depression, Anxiety, and Stress Scale (DASS-21). Is an instrument designed to assess the negative emotional states of depression, anxiety, and stress [87,88]. The depression subscale is formed by questions 3, 5, 10, 13, 16, 17, and 21. Questions 2, 4, 7, 9, 15, 19, and 20 formed the anxiety subscale. The stress subscale was formed by questions 1, 6, 8, 11, 12, 14, and 18. The total scores categorize each subscale into three levels: mild, moderate, and severe. Finally, participants will be classified as no risk (<6) or risk (>6).

Academic achievement: To assess this variable, the grades of all subjects from the first academic semester will be considered and compared with those of the second semester (intervention). The rating scale in Chile is from 1 to 7, with 7 being the maximum rating.

Health-related Quality of Life: An instrument that measures the health-related quality of life of adolescents. It is a self-report instrument applicable to healthy and chronically ill children and adolescents aged 8 to 18 years [89]. The KIDSCREEN-27 measures five dimensions: physical well-being (5 items), psychological well-being (7 items), autonomy and parents’ relations (7 items), peers and social support (4 items), and school environment (4 items) (Cronbach’s alpha = 0.89).

Perception of environmental urban features: Neighborhood Environment Walkability Scale (NEWS). A self-report measure that has recently been developed to assess perceived neighborhood environmental attributes related to physical activity. The full version was designed and validated among North American youth. Subsequently, it was translated into other languages and has been used in Spain [90,91]. It is composed of items that collect information on residential density, access to services, connectivity, walking and cycling infrastructure, neighborhood aesthetics, neighborhood safety, and access to sports facilities.

Physical activity barrier: The short scale of perception of physical activity barriers in adolescents will be used [92]. It consists of 12 questions that collect the self-perception of the different items included in the scale. Each item will be evaluated using a 5-point Likert response scale, where 1 means strongly disagree and 5 means strongly agree (Cronbach’s alpha = 0.80).

Active Transportation: A questionnaire that includes questions on reasons for choosing a particular school, transportation habits to school, motivations and barriers to walking and bicycling to school, perceived neighborhood environment, health behaviors, perceptions of driving, and use of information and communication technology. This questionnaire is based on the PACO Project [93].

Physical activity level (subjective): This will be assessed using the Physical Activity Questionnaire for Adolescents (PAQ-A) [94]. It consists of nine questions that evaluate different aspects of physical activity performed by adolescents in the previous 7 days, using a 5-point Likert scale for each question. It reports information on the intensity, frequency, and duration of each activity performed. The overall result gives a score from 1 to 5, where a higher score indicates a higher physical activity level. Participants will complete the Spanish version (ICC = 0.71).

School questionnaire: Based on the questionnaire used in the Spanish ALADINO study [95]. It includes date of birth, sex, place of residence, sports experience, grade, date and time of measurement, clothes worn at the time of measurement, and name and address of the school.

Parents’ self-perceived physical fitness: using the International Fitness Scale (IFIS) questionnaire [96]. It has a Spanish version, and there are studies published in Chile that use it [97]. This questionnaire is composed of five Likert-type questions that describe the perception of general fitness, cardiorespiratory fitness, muscle strength, speed agility, and flexibility (Kappa = 0.45).

Parents’ Physical Activity Level: Global Physical Activity Questionnaire (GPAQ) designed by WHO. It incorporates information on physical activity participation and sedentary behavior in three settings: activity at work, activity while moving, activity during leisure time, and activity at work [98].

Cultural, social, and political context: School Questionnaire (Directors): Includes questions related to school physical activity policy, infrastructure, sports equipment, educational mission and vision, food received at school, and access to food during school hours.

Covariates: familial socioeconomic status, perception of environmental urban features parent’s and physical fitness parent’s.

### 2.8. Statistical Analysis

The Statistical Package for the Social Sciences (SPSS) 25.0 will be employed for data analysis. Descriptive statistics will be conducted to derive summary and dispersion measures of the data. Subsequently, the variables will be subjected to Shapiro–Wilk tests to assess normality and Levene’s tests to evaluate homogeneity of variance.

#### 2.8.1. Intention-to-Treat Analysis

The statistical analysis plan is outlined as follows: Following verification of the effectiveness of randomization, mixed regression models will be employed, with each outcome variable serving as the dependent variable and interventions treated as fixed effects (1 = EG and 0 = CG). Models will be adjusted for baseline values, age, and classroom. Absolute differences in variable changes between baseline and final measurements will be reported as results. For dichotomous dependent variables, odds ratios will be calculated. Sensitivity analyses will involve multiple imputation techniques to address missing measurement values. Five databases will be imputed and analyzed in parallel. In the event of imbalances threatening group comparability, propensity scores will be utilized to control for covariate imbalances. Analyses will be conducted from an intention-to-treat perspective, where subjects remain in the analysis group (EG or CG) to which they were originally assigned, regardless of their intervention compliance. The results will be considered statistically significant at *p* < 0.05.

#### 2.8.2. Analysis by Protocol

Only participants who have attended at least 85% of the training sessions will be included in this analysis.

## 3. Discussion

The MOV-ES Project represents an extension of existing international projects assessing the effects of active transport interventions. However, while most of these study protocols are tailored to the characteristics of high-income countries, ours stands as the first in a Chilean and Latin American context. Furthermore, given the lack of specificity, updates, and scarcity of objectively measured epidemiological data on physical activity in Chile, there is a pressing need to develop monitoring and intervention initiatives for physical activity, active transportation, and the school environment based on Sallis et al.’s ecological model [4,99,100]. The efficacy of systematically assessed physical activity has been validated in various studies, demonstrating its impact on health promotion, disease prevention, influence on public policies, and augmentation of physical activity levels in the population [101,102,103,104].

Objective data regarding post-pandemic (for COVID-19) physical activity levels among Chilean adolescents could significantly inform public health decision-making and contribute to the formulation of educational and urban policies that benefit the entire educational community [105]. Furthermore, these results could allow this methodology to be included in national epidemiological studies, such as the National Health Survey—an essential step, given existing weaknesses in this area within studies based on the ecological model [90]. This represents a key strength of our protocol study. Additionally, our protocol study will enhance understanding of the impact of active transport on various health parameters, including body composition, cardiorespiratory risk, physical fitness, mental well-being, and cognition. Regardless of the outcomes, our findings will hold clinical and public health significance, shedding light on the health implications of transportation choices [20,22,23,24]. Ultimately, our results will pave the way for future research endeavors, addressing a topic that currently lacks robust empirical evidence [49]. The findings of our protocol study have the potential to be extrapolated to other cities sharing similar characteristics, serving as a foundational platform for future studies in this area across broader population demographics. This is particularly significant given Chile’s educational and urban landscape, which differs from that of developed countries. Interventions of this nature within such a context hold promise for enhancing educational processes. Notably, a key distinction is that in Chile, the selection of educational establishments by families is not determined by proximity to the home, posing a significant barrier to active transportation—a factor that warrants specific consideration and intervention strategies.

As previously noted, studies in the field of active transportation in Chile are still in their nascent stages, underscoring the importance of tailoring interventions to align with the country’s unique circumstances. Our protocol study focuses on first-year high school students, a demographic of particular concern given the documented low physical activity levels within this age group [6,7]. In addition, a recent systematic review confirms the need to evaluate the effectiveness of active transportation, specifically within this age cohort [106]. This targeted approach aims to address critical gaps in understanding and intervention implementation within the Chilean context.

Building upon the aforementioned considerations, the MOV-ES Project targets both the school community and the residential environment surrounding the educational institutions under study, with the aim of providing data recognized as key indicators of educational quality [107]. Recognizing the multifaceted nature of physical activity engagement and transportation choices, influenced by social, cultural, environmental, normative, and familial contexts, our protocol study endeavors to identify predictive factors, barriers, and beliefs pertinent to physical activity behaviors among adolescents and parents [108]. Utilizing the framework delineated by Sallis et al.’s ecological model, which encompasses the domains of occupation, transportation, recreation, and home, we aim to develop and implement an effective intervention strategy that positively impacts Chilean schoolchildren. This comprehensive approach seeks to address the complex interplay of factors shaping physical activity engagement and transportation choices within the local context [46].

The MOV-ES project has the potential to serve as a valuable public utility tool, offering insights and recommendations to key stakeholders such as the Chilean Ministry of Education, the Ministry of Housing and Urban Planning, and the Ministry of Sports. By tailoring interventions to reflect the unique realities of the Chilean context and advocating for pedagogical and regulatory adaptations within school environments, this study protocol aims to prompt reflection and foster positive changes. One of the key strengths of this intervention lies in its cost-effectiveness, ease of implementation, and replicability, making it feasible for widespread adoption. Ultimately, the implementation of such interventions holds promise for improving the overall living conditions of schoolchildren and fostering healthier lifestyles within the community.

The impact of the study protocol (MOV-ES Project) is anticipated to extend beyond merely enhancing the physical health of high school students, with potential implications for the broader school community, notably by alleviating congestion within school environments. These outcomes are poised to furnish invaluable insights for the design and implementation of future nationwide active transportation interventions aimed at bolstering physical activity levels among adolescents [75,109]. Given the active involvement of decision-makers, the findings will be pertinent to local contexts and practices, thereby facilitating evidence-based decision-making [4]. The comprehensive nature of the study stands to enrich scientific understanding regarding the myriad factors influencing physical activity engagement among adolescents. Consequently, stakeholders such as the Chilean Ministry of Education, regional educational administrative bodies (DAEMs), municipalities, and educational institutions will be empowered to formulate targeted public policies conducive to promoting physical activity and cultivating healthy habits during the school years. By aligning initiatives with the actual needs and environmental dynamics of schoolchildren, these efforts aim to optimize the provision of physical activity and sports programs [75]. This strategic approach is underpinned by quality indicators delineated by the Education Quality Agency, ensuring that interventions are attuned to the imperatives of educational excellence [107].

Among the possible limitations of the study protocol are: (i) the difficulty of accessing the selected schools through randomization; (ii) the impossibility of some participants to complete the contemplated sessions; (iii) the impossibility of having continuity in the 16 weeks of intervention due to events external to the research team; and (iv) parents’ refusal to authorize their children to take active transportation.

The strengths of the MOV-ES Project are: (i) it represents an extension of existing international projects that evaluate the effects of active transportation interventions contextualized to Chile; (ii) it will provide objective data on physical activity and active transportation in Chile; (iii) the effectiveness of the intervention will be measured using the Ecological Model of Physical Activity; and (iv) it has the potential to serve as a valuable public utility tool, offering ideas and recommendations to different government institutions.

## 4. Conclusions

This study protocol outlines comprehensive research into the impact of an active transportation education program, grounded in the ecological model, on the physical and mental well-being of high school students. Our hypothesis posits that implementation of such a program will yield a reduction in mean body fat percentage alongside enhancements in executive function, encompassing inhibition, cognitive flexibility, and working memory, among high school students. Should our intervention demonstrate efficacy, there is potential for its integration within both public and private physical activity initiatives targeted at Chilean high school students. This underscores the importance of evidence-based interventions in fostering the health and well-being of adolescent populations.

## Figures and Tables

**Table 1:** Progression for MOV-ES intervention.

Units	Month	Weeks	Location
Unit I: benefits of physical activity on health and healthy habits in schoolchildren	1	1–4	Classroom
Unit II: Active Transportation: Experiences from other countries	2	5–8	Classroom
Unit III: Analysis of the environmental characteristics of the school environment of each establishment	3	9–12	Inside the School
Unit VI: Road safety for pedestrians and cyclists. This last topic will be broken down considering analysis and prevention of accidents with automobiles, pedestrian regulations, signaling, and cyclist safety	4	13–16	Outside the school

**Table 2:** Variables and instruments of the Ecological Model-Based Active Transport Education intervention.

Variable	Dimension
Body composition	INTRAPERSONAL FACTORS
Excutive Functions	
Body composition	
Physical activity level (objective)	
Physical fitness	
School questionnaire	
Health-related Quality of Life	
Perception of environmental urban features	
Physical activity level (subjective)	
Adherence to physical activity	
Active Transportation	
Mental well-being: stress, anxiety, depression	
Academic achievement	
Parents’ self-perceived physical condition	INTERPERSONAL FACTORS
Parents’ Physical Activity Level	
Cultural, social, and political context: School Questionnaire (Directors)	PHYSICAL ENVIRONMENT FACTORS

## Data Availability

Data are contained within the article.
